# APEX2- tagging of Sigma 1-receptor indicates subcellular protein topology with cytosolic N-terminus and ER luminal C-terminus

**DOI:** 10.1007/s13238-017-0468-5

**Published:** 2017-09-19

**Authors:** Timur Mavylutov, Xi Chen, Lianwang Guo, Jay Yang

**Affiliations:** 1 Department of Anesthesiology, University of Wisconsin SMPH, Madison, WI 53705 USA; 2 Department of Surgery and Physiology & Cell Biology, Ohio State University, Columbus, OH 43210 USA

**Dear Editor,**

Deciphering the role of any protein in a cell requires knowledge of the structure, subcellular localization, and the topological orientation of the protein within the relevant cells. The Sigma-1 receptor (S1R) largely localized to the endoplasmic reticulum (ER) serves as a pluripotent intracellular signaling molecule with diverse roles in the cell including ion channel modulation, stress signaling, and transcriptional regulation (reviewed in Su et al., 2016), however, the basic topology of this protein within a cell remains controversial. The topology of the N- and C-termini of the S1R based on the antibody accessibility technique was reported with both N- and C-termini facing the cytosol (Aydar et al., 2002), while a later report (Hayashi and Su, 2007) suggested that both termini were facing the ER lumen. These topological conclusions assumed a protein structural model with two transmembrane (TM) domains based on the hydrophobicity property of the protein. More recently, the study resolving the crystal structure of the S1R reported the protein to possess a single TM domain with a short N-terminus facing the ER lumen, while most of the protein bulk was located on the cytosolic side of the ER membrane (Schmidt et al., 2016) (Fig. 1A). Due to contradictory reports on the exact topology of the S1R within the ER membrane, we applied the ascorbate peroxidase 2 (APEX2) approach to provide a definitive answer to the S1R protein topology in the ER membrane. A unique feature of APEX2 is that it retains robust peroxidase activity even after strong fixation with 2% glutaraldehyde and brings about its utility as a tag for subcellular detection of proteins of interest with electron microscopy (EM) with clarity not attainable by the more conventional immuno-electron microscope technique (Rhee et al., 2013; Lam et al., 2015; Lee et al., 2016).

**Figure 1:**
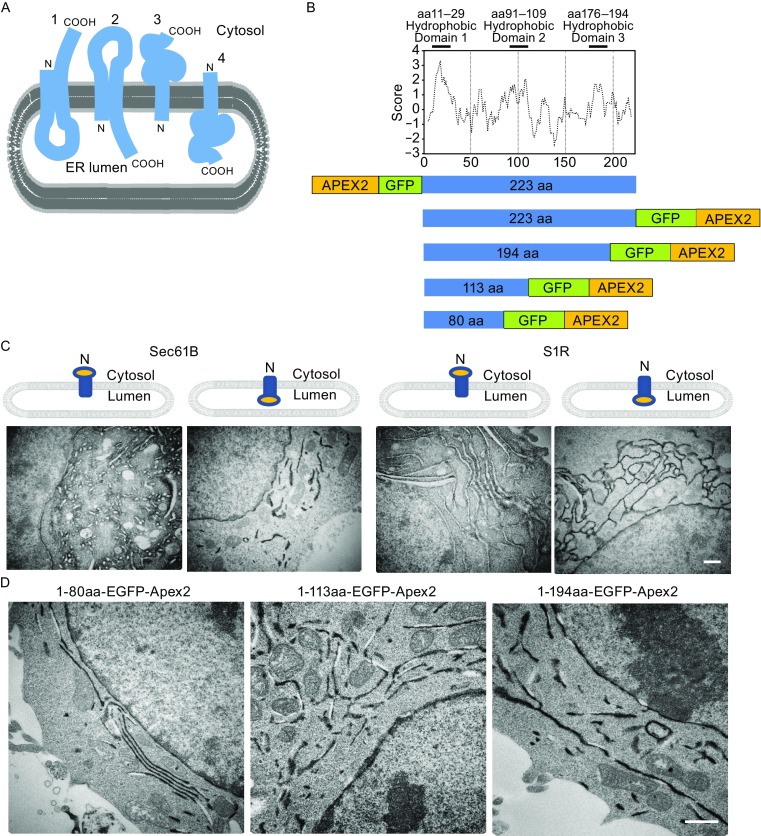
**Determination of S1R topology in the ER by APEX2-tagging**. (A) Three topologies of S1R in the ER membrane have been proposed. 1. Both N- and C-termini are cytosolic with two TM domains (Aydar et al., 2002), 2. Both N- and C-termini are luminal with two TM domains (Hayashi and Su 2007), 3. N-terminus in the lumen with one TM domain (Schmidt et al., 2016), and 4. A novel topology with N-terminus in the cytosol with one TM domain (current study). (B) Hydrophobicity plot of the 223-residue human S1R protein (top) with a cartoon of the hydrophobic potential TM domains indicated above. The putative first TM spans aa 11–29 and the second TM spans aa 91–109. The third relatively hydrophobic stretch of residues thought to possibly dip into the membrane spans aa 176–194. Positive score values are increasingly hydrophobic (http://www.web/expasy.org/protscale/). The cartoons below are the five GFP-APEX2 fusion S1R constructs (one N-tagged and four C-tagged) examined in the current study. The truncation constructs separate the major hydrophobic potential TM domains of the S1R protein. (C) Cartoon of the ER membrane spanning control Sec61B with the N-terminus facing the cytosol or the S1R indicating the location of the APEX2 tag. The images are EM photomicrographs of the respective constructs expressed in ND9/27 cells. The presence of “open” whitish appearance of the ER lumen indicates the lack of electron-dense reaction product in the ER lumen consistent with the APEX-tag facing the cytosol, while the “solid” blackish ER appearance inside the ER lumen indicates localization of the APEX-tag facing the ER lumen. (D) Similar experiment with expression of C-terminus-tagged S1R-truncation constructs. S1R 1–80aa spans the presumed first TM domain (left), S1R 1–113aa extends to just beyond the putative second TM domain (middle), and S1R 1–194aa spans the putative third hydrophobic domain (right) (see Fig. 1B). The solid blackish ER lumen indicates the presence of the APEX2-tag facing the ER lumen for all truncation constructs. Scale bar: A = 2 µm for upper panel, and 1 µm for lower panel; B = 1 µm

We created constructs encoding the full-length S1R with APEX2 attached at either the N- or C-terminus of the receptor (Fig. 1B). We also created Sec61B constructs, a one TM domain component of the ER translocon with an insertion topology in the ER with the protein N-terminus facing the cytosol (Dudek et al., 2015). Previous reports indicate that the tagging of Sec61B with APEX2 attached either at the N- or C-terminus does not alter the subcellular targeting to the ER or the topology of the protein inserted into the ER membrane (Lee et al., 2016). Since all constructs also contained green fluorescent protein (GFP) positioned between S1R and APEX2, fluorescence microscopy allowed detection of the expression of the fusion protein. Confocal imaging of ND7/23 cells co-transfected with the ER-localizing Sec61B-mCherry and N- or C-tagged S1R confirmed a direct overlap of the GFP and mCherry signal in the ER, indicating no mistargeting due to epitope-tagging (Fig. S1A).

When transfected into cells, the control Sec61B protein was detected in the ER as expected. EM imaging confirmed accumulation of the electron-dense precipitate in the cytosol when the APEX2-tag was attached to the N-terminus of Sec61B, while the APEX2-tag attached to the C-terminus of Sec61B resulted in electron density in the ER lumen (Fig. 1C, left two panels). An analogous experiment with full-length S1R resulted in detection of electron-dense precipitates in the cytosol when APEX2 was attached to the N-terminus of the receptor, while luminal electron-density was detected with APEX2 attached to the C-terminus of the receptor (Fig. 1C, right two panels). This supports the conclusion that the N-terminus of the full-length S1R faces the cytosol while the C-terminus faces the ER lumen.

To probe for the possible presence of TM domains within the S1R protein, we created three different S1R truncations with APEX2 attached at the C-terminus. The three truncation constructs encoded for amino acids 1–80, 1–113, and 1–194 demarcated the boundaries of potential TM domains based on the hydrophobicity plot of the protein. If a TM domain is present, the C-terminus of the truncation construct encompassing such a domain should localize to the opposite side of the ER membrane. EM analysis of the cells expressing these truncation constructs demonstrated localization of the C-terminus of all the constructs in the ER lumen consistent with the interpretation that only one TM domain exists between amino acids 1–80 (Fig. 1D).

A silver nitrate and gold chloride modification of the technique allows higher resolution detection of the APEX2-catalyzed reaction product as electron-dense nanogold precipitates, rather than as diffuse diaminobenzidine precipitates localizing outside or inside the ER lumen (Mavlyutov et al., 2017). Enhanced-resolution EM images of ND7/23 cells expressing N- or C-terminus-tagged Sec61B and S1R showed the same pattern of localization with the expected cytosolic punctate nanogold precipitates for N-tagged Sec61B and S1R. The C-tagged Sec61B and S1R both showed ER luminal localization of the precipitates. The protein topology revealed by the enhanced higher resolution imaging was identical to the pattern obtained without intensification, although electron-dense signals appeared to be sharper and more intense with this technique (Fig. S1B).

We wanted to confirm the validity of the S1R topology determined in transfected ND7/23 cells in primary neurons. We have an ongoing interest in the role of S1R in pathological pain and therefore decided to target the dorsal root ganglion (DRG) as our neuronal target where S1R is abundantly expressed (Mavlyutov et al., 2016). Intrathecal administration of adenoassociated virus (AAV) 2/5 or 2/8 vector allows selective transduction of DRG neurons (Storek et al., 2008; Mason et al., 2010). We created AAV2/5 expressing GFP alone or S1R-GFP-APEX2 (full-length) and administered the vector by an intrathecal injection. The lumbar spinal cord and DRG were isolated 4 weeks after virus administration, and a robust GFP fluorescence was detected in the central projections of the DRG neurons into the spinal cord proper and the DRG neurons (Fig. 2A and 2B). When the S1R-GFP-APEX2 virus was administered, the GFP fluorescence was restricted to the DRG soma consistent with the soma-restricted expression of the endogenous S1R as reported previously (Mavlyutov et al., 2016). The GFP-fluorescence in the DRG soma was punctate in appearance, excluding the nucleus, consistent with the mostly ER-limited expression of the S1R. The biotin-phenol reaction of the DRG sections demonstrated a robust streptavidin-Cy3 signal, completely overlapping the GFP fluorescence (Fig. 2C–E). EM analysis of AAV-transduced DRG neurons demonstrated electron-dense precipitates most readily detected in the ER and nuclear membranes, and a closer inspection of the images indicated precipitates in the ER lumen and internuclear membrane space (Fig. 2F and 2G). The topology of the S1R expressed in the DRG neurons, *in vivo*, is consistent with that detected in transfected ND7/23 cells with the C-terminus of the protein facing the ER lumen. Electron-dense precipitates indicating localization of the S1R could be found at the mitochondria-associated membrane of the ER (Fig. 2H and 2I) (Hayashi and Su, 2007) and the subsurface cisterns (Fig. 2J) (Mavlyutov et al., 2010; Mavlyutov et al., 2015b), but not at the plasma membrane.

**Figure 2:**
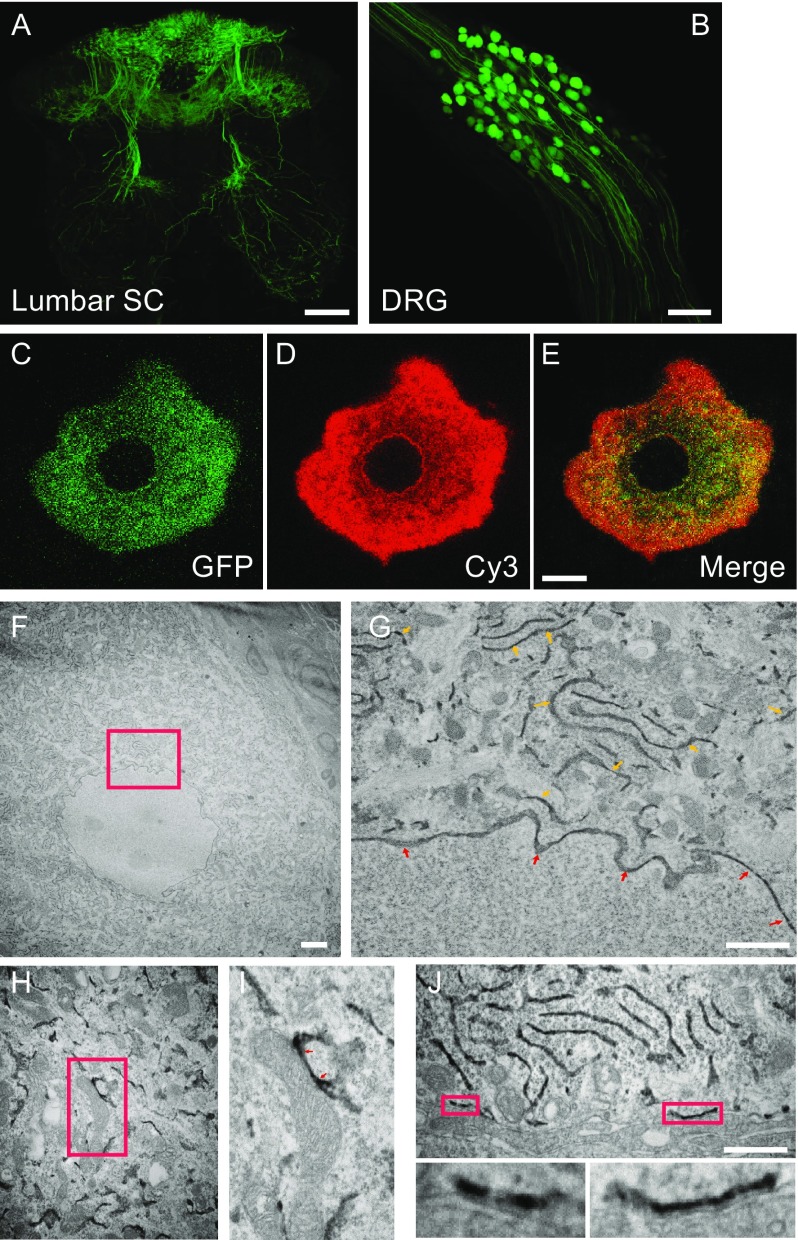
**Topology of S1R in primary DRG neurons transduced with AAV2/8**. (A) Fluorescent image of a rat lumbar spinal cord section transduced with AAV2/8-EGFP 4 weeks earlier. Dorsal (top) and ventral (bottom). (B) A stacked confocal image of a lumbar DRG from the same rat. Transduced EGFP-expressing DRG neuronal cell body and the neurites extending both proximally (top) and distally (bottom) can be seen. (C–E) A fluorescent image of a single neuron from a rat transduced with AAV2/8 (S1R-EGFP-APEX2; C-terminus-tagged full-length S1R) 4 weeks earlier. The GFP-image shows successful expression of the EGFP reporter, and the Cy3-image shows the same neuron probed with streptavidin-Cy3 after reacted the DRG section with H_2_O_2_ and biotin-phenol for proximity-labeling. The merge image shows a complete overlap of the two signals. (F and G) An EM image of a DRG neuron from the same rat. ER (yellow arrows) and nuclear membrane (red arrows) of the magnified area within the red box show electron-dense precipitates within the ER lumen and the inter-nuclear membrane space. (H and I) Electron-dense precipitation can be observed in ER associated with mitochondria (i.e., mitochondria-associated membrane) (red arrows). (J) Electron-dense precipitation is observed in subsurface cisterns, but not in the plasma membrane (PM). Lower panels are magnified images of subsurface cisterns outlined in the red box in the upper panel. Yellow arrows point to the plasma membrane. Scale bar: A = 200 µm; B = 100 µm; C–E = 10 µm; F = 2 µm; G = 1 µm; H = 1 µm; I = 0.5 µm; J = 0.5 µm

The structure of the S1R was solved after overexpression of the FLAG-tagged S1R in baculovirus, affinity purification of the protein, reconstitution of the receptor into lipidic cubic phase, and crystallization by the hanging drop technique (Schmidt et al., 2016). The technique allowed accurate determination of the complex trimeric crystal structure of the S1R with likely larger scale oligomeric assemblies, but it is unclear how the assignment of the luminal vs. cytosolic orientation within the cellular context was made in an artificially reconstituted membrane. We believe the topological assignment of the N-terminus of S1R facing the cytosol and the C-terminus facing the ER lumen, based on our electron microscopic evidence of the APEX2-tagged protein expressed in a cellular biological context, better reflects the true S1R topology.

Limitations of the imaging aspect of the current study include: 1. Potential mistargeting of the S1R due to tagging with GFP-APEX2, 2. Possible misfolding of the truncated S1R leading to mistargeting, and 3. Misidentification of cells selected for EM imaging, especially for constructs where the expected electron-dense precipitates reside outside of the ER lumen. Subcellular mistargeting of a protein could occur from placement of an epitope-tag on either terminus of the S1R. In fact, placement of an N-terminus EYFP tag has been reported to mistarget this fusion protein from a lipid-rich ring-like structure in the ER (Hayashi and Su, 2003). However, our control experiments (Fig. S1) did not find any evidence for mistargeting of the GFP-APEX2-tagged S1R, at least at the confocal light microscope level. The conclusion that the N-terminus of the S1R faces the cytosol rests on the observation that the ER lumen is devoid of the electron-dense precipitates in EM images. Such lack of precipitation in the ER lumen could be because of a technical reason that the cell selected for EM examination was not transfected. However, such a technical error is unlikely because a side-by-side comparison of untransfected and those transfected with a cytosolic-localizing construct clearly shows a discernible presence of a cytosolic haze for the latter (data not shown). In addition, the enhanced processing of the cells using silver nitrate and gold nanoparticle clearly shows a cytosolic localization of particles for the N-terminus-tagged S1R with little chance of a false-negative conclusion.

The newly identified S1R topology with the bulk of the protein residing in the ER lumen with only a short segment facing the cytosol has functional implications. Many of the experimentally confirmed S1R interacting proteins such as BiP, IRE1, IP3R, ankyrin, emerin, and RanBP2 (see Fig. 1 in Su et al., 2016) are ER- or nuclear membrane-resident proteins. Furthermore, the large number of plasma membrane-resident ion channels and signaling molecules reported to interact with the S1R was puzzling given the very limited presence of S1R in the plasma membrane. However, since the plasma membrane-targeted proteins necessarily traverse the ER during subcellular sorting to the final destination, both the S1R with bulk of its protein in the ER lumen and the various plasma membrane proteins reside in the same ER subcellular compartment, providing a common physical location for potential protein: protein interaction to occur. In contrast, the short N-terminus of S1R facing the cytosol is likely to render interactions with truly cytosolic localizing partner proteins challenging.

In summary, the topology of the S1R identified in the present study is consistent with the crystal structure proposed by Schmidt et al., (2016), except that the N-terminus of the protein faces the cytosol.

## Electronic supplementary material

Below is the link to the electronic supplementary material.

Supplementary material 1 (PDF 3584 kb)
