# Supplementary material for: APEX2- tagging of Sigma 1-receptor indicates subcellular protein topology with cytosolic N-terminus and ER luminal C-terminus
Source: Protein Cell. 2017 Sep 19;9(8):733–7. doi: 10.1007/s13238-017-0468-5 (PMC6053353; doi:10.1007/s13238-017-0468-5)
Supplement: Supplementary file 1 — Supplementary material 1 (PDF 3584 kb) [file 13238_2017_468_MOESM1_ESM.pdf]

Supplemental Material

Material and Methods:

Animals. The study was approved (Protocol M02569) by the local institutional animal care use committee, and all rats were treated in accordance with published NIH standards. Male Sprague-Dawley rats were purchased from Envigo Lab (Envigo, Madison, WI, USA), maintained on a normal 12-h dark/light cycle, and handled in accordance with animal care and use guidelines of the University of Wisconsin, Madison. Animals were maintained on a 4% fat diet (HarklandTeklad, Madison, WI, USA) with food and water available *ad libitum*.

Intrathecal injections: Rats were deeply anesthetized by isoflurane and 20 µl of AAV2/5-S1R-green fluorescent protein. (GFP)-APEX2 or AAV2/5-GFP (~6.0 x 10^{12} vg/ml) (ViGene Biosciences, Rockville, MD) were intrathecally delivered between the L4 and L5 spinal segments of anesthetized rats using a 30-gauge needle. For electron-microscopic analysis of transgene expression in DRG, animals were euthanized and intracardially perfused with 2% glutaraldehyde in 0.1 M phosphate buffer (PB) 4-6 weeks after intrathecal virus injection.

Molecular Constructs. The full-length and truncated human S1R cDNA (NM 005866, Origene, Rockville, MD) (**see Figure 1B**) and Sec61B (NM 006808, Origene) were PCR-amplified using Pfx polymerase (Invitrogen, Carlsbad, CA). The fragments required for the GFP-APEX2 C-terminus tag and APEX2-GFP N-terminus tag, including the linker sequence GSGSTSGSG between the GFP and APEX2, were amplified from the connexin43-GFP-APEX2 pcDNA3 vector (a gift from Alice Ting, Addgene plasmid # 49385). For GFP-APEX2 tagged to C-terminus of S1R or Sec61B, stop codon of S1R and Sec61B was replaced with a Mlu I restriction enzyme recognition sequence (ACGCGT) and fused to the GFP-APEX2 sequence. For constructs with APEX2-GFP positioned on the N-terminus of S1R and Sec61B, the start codon was replaced with the Mlu I sequence and fused to the 3' side of APEX2-GFP. All cDNAs were placed in the multiple cloning site (between EcoR I and Not I or Nhe I and Not I) of the pCI/neo eukaryotic expression vector (E1841, Promega, Madison, WI). All PCR products were sequenced to confirm the fidelity of the amplification. The primers used for the various PCR amplification are listed in **Supplemental Table I**. For the creation of AAV2/5, cDNA encoding GFP or S1R-GFP-APEX2 was subcloned into an CMV-driven pAAV-MCS shuttle vector (VPK410, Cell Biolabs, San Diego, CA), expression confirmed by a Western blot, and the plasmids submitted for viral rescue to create the AAV5 or 8 pseudo-typed viral particles.

Cells. ND7/23 cells (#92090903, Sigma-Aldrich, St. Louis, MO) were maintained in DMEM medium supplemented with 10% fetal bovine serum and penicillin/streptomycin. Cells were

seeded on poly-l-ornithine-coated glass coverslips inside the wells of a plastic 24 well plate. Cells were transfected with plasmids using Lipofectamine 2000 according to the supplied protocol. About 24 hrs later, transfection cells were incubated with 500 µM biotin-phenol (#CDX-B0270, Adipogen, San Diego, CA) in complete DMEM medium for 30 min in a volume of 0.5 ml/well. Precisely 30 minutes after incubation with biotin-phenol, H_2O_2 was added to wells to achieve a final concentration of 1 mM for 1 minute. The media was quickly aspirated and cells washed three times with the quencher solution (1x DPBS, 10 mM sodium azide, 10 mM sodium ascorbate, 5 mM Trolox), and cells were fixed for 20 min with 4% formaldehyde in 1x DPBS for signal detection with fluorescence microscopy. A cartoon (**Figure 1C**) illustrates the expected localization of electron-dense reaction products in the cytosol or the ER lumen depending on the location of the APEX2-tag.

Fluorescence microscopy. After fixation cells were washed three times (2-3 min each time) in DPBS, quenched for 3 min in 20 mM glycine, washed and permeabilized with 0.1% Triton X100 for 20 min, blocked in 1% BSA for 20 min, and incubated for one hour with streptavidin conjugated to Cy3 fluorophore (#405215, Biolegend). Coverslips with cells were rinsed three times, poststained with DAPI nuclear stain, rinsed again, and mounted onto a glass slide in Prolong Diamond mounting medium (P36930, Thermo Fisher). The slides were left in the dark overnight and sealed with nail polish (Electron Microscopy Sciences, Hartfield, PA, USA). Images were taken with a Leica confocal microscope equipped with a green 488 Argon laser and a red 561 nm DPSS laser through an 60X water-immersed objective. Images were processed using the ImageJ program.

Electron microscopy. 24 hrs after transfection, cells or vibratome sections for rat primary tissues, were fixed with 2% glutaraldehyde in 1x DPBS (Dulbecco modified phosphate buffered saline) for 20 minutes, rinsed three times, and quenched in 20 mM glycine. Samples were rinsed three times, and a solution of 0.5 mg/ml DAB (D12384, Sigma Aldrich) with 10 mM H_2O_2 in 1x DPBS was added to develop brown precipitates of DAB polymer for 7-10 min. Coverslips with cells and vibratome slices of the tissues were rinsed and stained with 2% aqueous OsO_4 for 30 min. Samples were rinsed and dehydrated in increasing concentration of ethyl alcohol followed by propylene oxide, mixture of propylene oxide and EPON resin, and followed by two changes of pure EPON resin. Then samples with cultured cells on coverslips were polymerized overnight in an oven at 60 °C. The samples of vibratome sections were polymerized similarly, but between two Teflon-coated glass slides. The glass was dissolved in hydrofluoric acid and samples were carefully rinsed to remove the residual glass. Areas with cells were cut with a jeweler saw and glued to the resin block. For vibratome sections, areas with tissue were

trimmed with a razor blade and glued to an empty EPON block. All samples were cut at 70 nm on an ultramicrotome. Thin sections were poststained with 8% depleted uranyl acetate for 10 min, and viewed and imaged with a Phillips CM120 STEM electron microscope.

For silver and gold particles-enhanced imaging, the following modifications were done to the procedure: After the development of brown DAB precipitates prior to osmification, samples were additionally post-fixed in 2% glutaraldehyde followed by washing three times in 100mM Tris-maleic acid, (pH7.4). Electron-dense polymer of DAB was further intensified by a German Silver Intensification mixture (0.52% hexamethyltetramine, 0.04% silver nitrate and 0.04% sodium tetraborate all in 100 mM Tris-maleic acid for 10 min at 60 °C in the dark (the components must be sequentially dissolved in nanopure water in the dark prior to adding the buffer). Samples were sequentially washed in nanopure H_2O and 0.01M PBS, followed by osmification in 1% OsO_4 for 30 min. The dehydration/embedding process was as described above.
For all EM experiments, 5-6 serial thin sections in a ribbon cut from an EPON block were mounted on a grid, scanned at low power to identify areas of dark electron dense precipitates, and selected areas imaged at a higher magnification from at least 4 cells. A minimum of 3 biological replicate experiments were performed on ND7/23 cells and DRG samples were obtained from 2 rats each injected with the 2 viral vectors.

Supplemental Figure 1. A. *N- or C-terminal tagging with GFP-APEX2 has no effect on S1R localization to the ER.* Confocal images of ND7/23 cells transfected with **A.** APEX2-GFP- (N-tagged) or **B.** GFP-APEX2- (C-tagged) S1R and both co-transfected with Sec61B-mCherry as a control ER marker. The images are GFP (left), mCherry (middle), and merged (right). Bar= 5 μm. **C.** *Gold/silver enhanced EM images of ND9/23 cells expressing the indicated constructs*. After silver/gold enhancement of DAB precipitate APEX2 attached to N-terminus of S1R appeared to face to the cytosol, while APEX2 attached to C-terminus was detected in the ER lumen. First two panels show control protein Sec61B with APEX2 at N-terminus (signal in the cytosol) and at C-terminus (signal in the ER lumen). APEX2 position at N-terminus of S1R (third panel) has gold dot signal detected on the cytosolic part of the ER, while APEX2 positioned at C-terminus of S1R (fourth panel) has signal detected in the ER lumen. Scale bar: upper panel = 0.5 µm, and lower panel = 0.25 µm.

Supplemental Table 1. *Primers used for PCR amplification of the indicated fragments.* All PCR amplifications were done with pairs of primers except for the APEX2-GFP, which required a 3-

piece ligation of the two PCR products Nhe-APEX2-EcoRI, EcoR1-GFP-Mlu, and the vector. Introduced restriction enzyme sites are noted in lowercase and the sequence encoding the flexible linker between the APEX2 and GFP is denoted in italics.

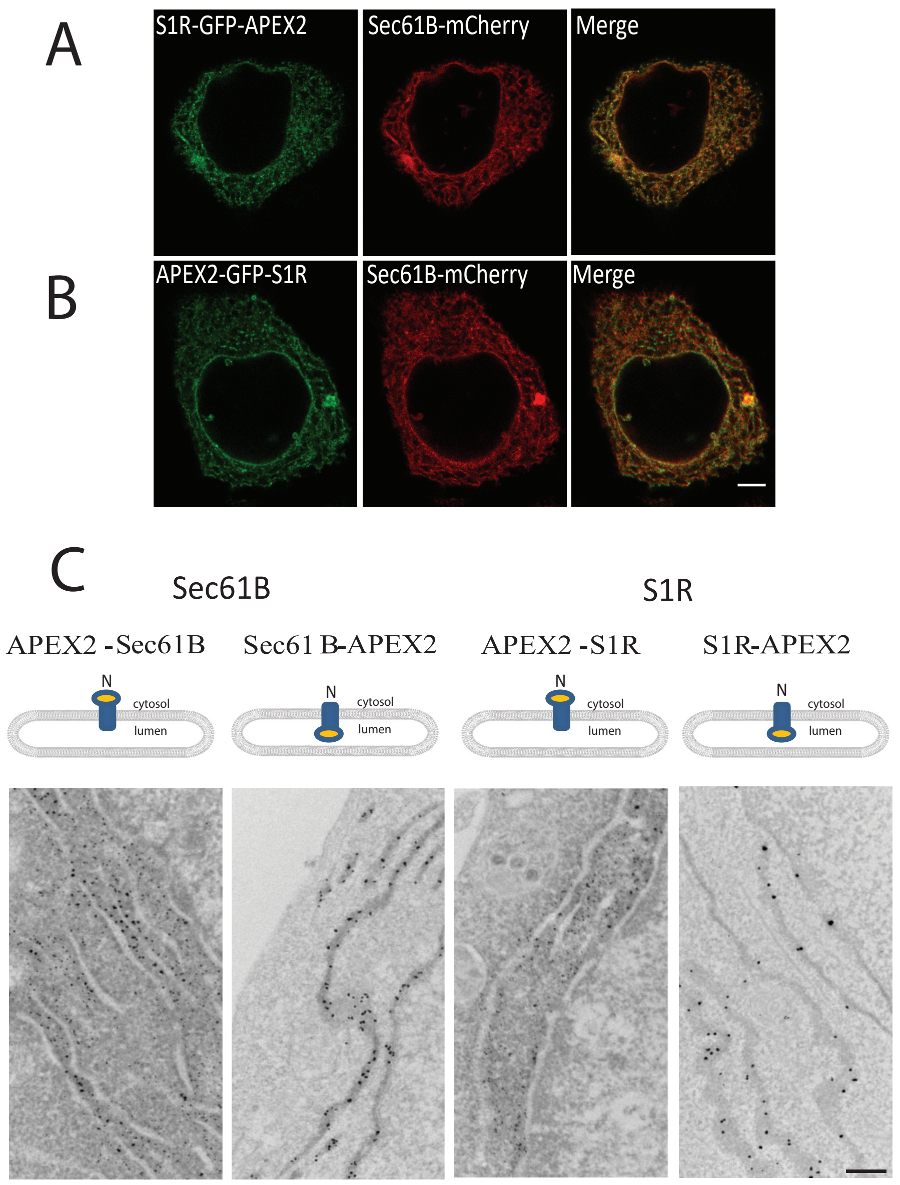

Supplemental Figure 1

Supplemental Table I:

Description	PCR Primers (5' to 3')
Apex2-GFP-Mlu-Fwd	acgcgtGTGAGCAAGGGCGAGGAGCTGTTCACC
Apex2-GFP-Not-Rev	gcggccgcTTAGGCATCAGCAAACCCAAGCTCGGA
Apex2-Nhe-Fwd	gctagcATGGGAAAGTCTTACCCAACTGTGAGTGCT
Apex2-ER1-linker-Rev	*gaattcCCCGAGCCCGAGGTCGAGCCCGAGCCC*
	GGCATCAGCAAACCCAAGCTCGGAAAGCTT
GFP-ER1-Fwd	*gaattc*GTGAGCAAGGGCGAGGAGCTGTTCACCGGG
GFP-Mlu-Rev	acgcgtCTTGTACAGCTCGTCCATGCCGAG
S1R-Fwd	gaattcATGCAGTGGGCCGTGGGCCGGCGGTGG
S1R-aa80-Mlu-Rev	acgcgtCTGCAGCTCCTCGTCGGGCAGCACGTG
S1R-aa113-Mlu-Rev	acgcgtGGAGCCCAAGGCGGTGCCGAAGAGCAG
S1R-aa194-Mlu-Rev	acgcgtCTGGGTGCTGAAGACAGTGTCGGCCAG
S1R-aa223-Mlu-Rev	acgcgtAGGGTCCTGGCCAAAGAGGTAGGTGGT
S1R-Mlu-Fwd	acgcgtCAGTGGGCCGTGGGCCGGCGGTGG
S1R-stop-Not-Rev	gcggccgcTCAAGGGTCCTGGCCAAAGAGGTAGGT
Sec61B-Nhe-Fwd	gcgagcATGCCGGGTCCAACGCCCAGTGGCAC
Sec61B-Mlu-Rev	acgcgtTCGCGTGTACTTGCCCCAAATGT
Sec61B-Mlu-Fwd	acgcgtCCGGGTCCAACGCCCAGTGGCAC
Seg61B-stop-Not-Rev	gcggccgcTGATCGCGTGTACTTGCCCCAAA
